# Cultural adaptation and validation of the SESMEB scale for assessing the influence of social media on the eating habits of adolescents in the city of Doboj, Bosnia and Herzegovina

**DOI:** 10.3389/fnut.2026.1935743

**Published:** 2026-10-07

**Authors:** Dragana Stojisavljević, Marina Jotić Ivanović, Biljana Mijović, Maja Nikolić, Jelena Niškanović, Bojan Joksimović

**Affiliations:** 1 Department of Nutrition, The Public Health Institute of Republic of Srpska, Banja Luka, Bosnia and Herzegovina; 2 Department of Public Health, Faculty of Medicine, University of Banja Luka, Banja Luka, Bosnia and Herzegovina; 3 Department of Family Medicine, Primary Health Care Centre, Doboj, Bosnia and Herzegovina; 4 Department of Primary Health Care and Public Health, Faculty of Medicine, University of East Sarajevo, Foča, Bosnia and Herzegovina; 5 Department of Social Medicine, Faculty of Medicine, University of Niš, Niš, Serbia; 6 Department of Psychology, Faculty of Philosophy, University of Banja Luka, Banja Luka, Bosnia and Herzegovina; 7 Department of Preclinical Subjects (Pathological Physiology), Faculty of Medicine, University of East Sarajevo, Foča, Bosnia and Herzegovina

**Keywords:** adolescents, eating behavior, food marketing, SESMEB questionnaire, social networks

## Abstract

**Introduction:**

Marketing of foods high in fat, sugar and salt shapes the food preferences and intake of children and adolescents. The primary aim of this study was to culturally adapt and validate the Scale of Effects of Social Media on Eating Behavior (SESMEB) for assessing the influence of social media on the eating habits of adolescents in the Republic of Srpska, Bosnia and Herzegovina, and to examine whether this influence differs by sex, place of residence, nutritional status and school attended.

**Methods:**

A cross-sectional study was conducted in six secondary schools in the city of Doboj in May 2023 and included 362 adolescents attending the third and fourth grades. The SESMEB scale was cross-culturally adapted into Serbian through forward and back-translation, expert panel review and pre-testing. Internal consistency was assessed by Cronbach’s alpha and corrected item-total correlations, the factor structure by exploratory factor analysis, and test–retest reproducibility in a subsample of 14 respondents by the intraclass correlation coefficient (ICC). Group differences were tested by *t*-test and ANOVA.

**Results:**

The adapted scale showed good internal consistency (Cronbach’s alpha = 0.865), with corrected item-total correlations from 0.346 to 0.638; the data were suitable for factor analysis (KMO = 0.857; Bartlett’s test *χ*^2^ (153) = 2312.380; *p* < 0.001). Exploratory factor analysis identified three subscales accounting for 51.5% of the variance, which could not be clearly separated. Test–retest analysis showed no significant difference between administrations (*t* = 0.552; *p* = 0.591; ICC > 0.7). Mean scores for all 18 items denoted no perceived influence, with 55.0–76.8% of respondents reporting no effect on their food-related attitudes and practices. Girls scored higher than boys for following nutrition-related content (*p* < 0.05) and for the increase in the desire to eat (*p* = 0.048); differences also emerged by place of residence (*p* = 0.041) and school attended (*p* = 0.008; *p* = 0.004).

**Conclusion:**

Adopted version of the SESMEB scale is a reliable instrument for assessing the perceived effects of social media on adolescent eating behavior. Most respondents reported no such influence, even though this population is intensively exposed to digital marketing of energy-dense products, suggesting that self-report may underestimate the actual effect of that exposure.

## 1 Introduction

Unhealthy diets are among the leading global public health risks, contributing to all forms of malnutrition, including undernutrition, micronutrient deficiencies, and overweight, obesity and diet-related non-communicable diseases (NCDs). Unhealthy food and beverage marketing directed at children and adolescents increases their dietary intake and negatively affects their preference for foods high in fat, sugar and salt (HFSS)—that is, foods high in saturated and trans-fatty acids, free sugars and/or salt, usually highly processed products whose consumption is associated with adverse health effects (1). In parallel, the Internet has become widely accessible worldwide, and its influence on everyday life continues to grow; according to Internet World Stats, the number of Internet users reached approximately 3.7 billion (49.6% of the world population) by March 2017, an increase of 93.8% between 2000 and 2017 (2). Social networks are today among the most common online activities of young people. A youth study conducted in Bosnia and Herzegovina in 2018/2019 found that Facebook and Instagram were the most frequently used platforms, whereas Snapchat was not widespread, and concluded that the attitudes of young people are largely shaped by social networks and online sources of information (3). Similar patterns have been reported elsewhere: among 386 adolescents in Türkiye the most frequently used networks were WhatsApp (88.2%), Instagram (77.5%), YouTube (70.9%), TikTok (43.5%), Twitter (21.5%) and Facebook (18%) (4), while among American adolescents aged 13 to 17 years the most used platforms were YouTube (95%), TikTok (67%), Instagram (62%), Snapchat (59%), Facebook (32%), Twitter (23%), WhatsApp (17%) and Reddit (14%) (5).

In this study, exposure to social media is understood as contact with content published on social platforms and the effect of that contact on the views, attitudes, emotions and behavior of those who see it. In the context of eating behavior, two pathways are of particular relevance. The first concerns purchasing and consumption decisions: advertisements, promotional posts, influencer recommendations and favorable product reviews increase the likelihood that a particular food will be bought and consumed, and such content most often promotes highly processed, energy-dense products. The second concerns the shaping of social norms: repeatedly seeing particular eating behaviors among peers, celebrities and influencers, such as eating fast food, drinking sugar-sweetened beverages or following a particular diet, may lead adolescents to perceive these behaviors as common, socially accepted and desirable. Both pathways operate largely without the deliberate attention of the recipient, which is relevant for the interpretation of self-reported measures of influence.

Adolescence is a period in which young people develop new skills in their desire to become more independent, while remaining vulnerable due to lack of experience; during this period, decisions about food are particularly important because of their long-term health consequences (6). Because of the exponential growth in social media use over the last decade, nutrition and health experts, governmental and non-governmental organizations and health professionals increasingly try to use social media to strengthen healthy food choices and nutrition-related behaviors in adults, children and adolescents (7). At the same time, advertisements for energy-dense, nutritionally poor foods specifically target children and young people and pose a threat to their health. Children continue to be exposed to powerful marketing of HFSS foods, the consumption of which is associated with adverse health effects (7, 8). Such marketing is prevalent—on packaging, in settings where children gather (e.g., schools and sports clubs), during children’s viewing times and on children’s channels, in youth magazines and on social media—and uses many techniques designed to appeal to young audiences (8). Digital marketing is of particular concern because it facilitates engagement, which can amplify the marketing message and its overall impact (8). On the other hand, social media can also be used to encourage healthy lifestyles through interventions that help individuals adopt healthy eating habits (9). This constant infiltration of social media into everyday life makes research into its effects on nutrition-related behavior increasingly necessary. Considering impact of social media exposure on children nutritional habits, the primary aim of this study was to culturally adapt and validate the Scale of Effects of Social Media on Eating Behavior (SESMEB), previously developed and validated in Türkiye (10), for assessing the influence of social media on the eating habits of adolescents, and to determine whether social norms conveyed through social media influence the eating choices of adolescents in the Republic of Srpska, Bosnia and Herzegovina. Accordingly, the study addressed the following research question: how is the use of social networks related to the eating habits of adolescents, with particular reference to the shaping of dietary preferences, decisions concerning the consumption of specific products, and the perception of a healthy diet?

## 2 Methods

### 2.1 Study design

A cross-sectional study was conducted in six secondary schools in the territory of the city of Doboj in May 2023. A validated and culturally adapted online questionnaire for the purposes of this research, designed in Google Forms was used. Respondents received a QR code that allowed them access to the questionnaire and was limited to one access to avoid duplication of data or misuse. During 7 days, all respondents received a QR code and access to the questionnaire. Respondents who in the first phase of the examination gave their voluntary consent to participate in the retest received an identification code through which they were able to access the questionnaire again after 7 days via SMS message. In the retest phase, a total of 14 respondents filled out the online form of the questionnaire after 7 days. By re-filling the online form of the questionnaire, data were obtained for examining the reproducibility of the agreement of repeated measurements with the same persons, that is, the stability of the questionnaire over time.

### 2.2 Participants

This study enrolled adolescents in the third and fourth grades of secondary schools in the territory of the city of Doboj: (1) Medical school, (2) Economic school, (3) Gymnasium, (4) Traffic and electrical school, (5) Technical school, (6) Hospitality and trade school. Of the 562 students attending the final grades of these secondary schools, 402 (71.5%) provided written informed consent, given either by the students themselves or by a parent or guardian. Of these, 40 (10.0%) did not complete the questionnaire and were excluded from further analysis, leaving 362 completed questionnaires (64.4% of all eligible students) that constituted the final study sample. The criteria for inclusion in the research were: (1) written consent to participate in research for students over 18 years old or written consent of parents/guardians for students under 18 years of age.

### 2.3 Questionnaire

The anonymous SESMEB questionnaire (10) was used in the research, which was modified according to the needs of the research and included two parts.

The first part of the questionnaire covered Sociodemographic data (gender, date of birth, school type, place of residence (city, village or suburban settlement)), and self reported Anthropometric data (Body height, Body weight and Body mass index) Body height and body weight were self-reported by the participants. Body mass index was calculated as body weight in kilograms divided by body height in meters squared, and BMI-for-age *z*-scores were derived using WHO AnthroPlus software (WHO growth reference for children and adolescents aged 5 to 19 years) (11). Nutritional status was classified according to age and sex as thinness (below −2 SD), normal weight (−2 SD to +1 SD), overweight (above +1 SD to +2 SD) or obesity (above +2 SD).

The second part of the questionnaire is the Scale of Effects of Social Media on Eating Behavior (SESMEB), which consists of 18 statements whose answers are measured using a Likert scale from 1 to 5, where one means never and five means always. In this way, test takers can have a score of a minimum of 18, or a maximum of 90 points. A higher score is associated with a greater influence of social media on an individual’s eating behaviors.

The scale of the influence of social networks on the eating habits of high school students was defined based on the average score for the given question: (i) 1–1.8 has absolutely no effect; (ii) 1.81–2.60 does not affect; (iii) 2.61–3.40 neutral; (iv) 3.41–4.20 affects; (v) 4.21–5 absolutely affects.

These cut-off bands were defined *a priori* by the authors for the purposes of the present study and were not adopted from the original instrument.

For the SESMEB questionnaire, prior to the beginning of the research, consent for use was requested and obtained from the author, via e-mail.

#### 2.3.1 Transcultural adaptation of the SESMEB questionnaire

Cross-cultural adaptation of the SESMEB questionnaire followed a four-stage procedure. In the first stage (forward translation), the instrument was translated from English into Serbian by two independent translators who were native speakers of Serbian with a good command of English; the two versions were subsequently reconciled and synthesized into a single Serbian version. In the second stage (back-translation), this synthesized version was translated back into English by a third translator, a native English speaker who had not been given access to the original English instrument. In the third stage, an expert panel comprising specialists in primary health care and family medicine together with qualified translators reviewed all items; no item required further modification of the Serbian translation. In the fourth stage, the pre-final version was pre-tested on a sample of 20 secondary-school students, all of whom confirmed that the items were clear and comprehensible. The final Serbian version of the questionnaire was accepted on this basis.

### 2.4 Statistical methods

Statistical data processing was performed in IBM SPSS Statistics version 20.0 (IBM Corp., Armonk, NY, United States) and Microsoft Excel (Microsoft Office 2016, Microsoft Corp., Redmond, WA, United States). The data collected by the research were analyzed using the usual methods of descriptive and inferential statistics, i.e., methods of univariate and multivariate statistics. The results of the study of the influence of social networks on the eating habits of high school students are presented using descriptive and analytical statistical methods. From the methods of descriptive statistics, the following were used: measures of central tendency (arithmetic mean and median), measures of variability (standard deviation) for continuous variables and absolute frequencies and percentages for categorical variables. The internal consistency of the SESMEB instrument was assessed using Cronbach’s alpha coefficient and corrected item-total correlations. The suitability of the data for factor analysis was verified using the Kaiser-Meyer-Olkin (KMO) measure of sampling adequacy and Bartlett’s test of sphericity, after which the factor structure was examined by exploratory factor analysis. Test–retest reproducibility was assessed in the subsample of respondents who completed the questionnaire on two occasions, using the intraclass correlation coefficient (ICC), the paired-samples *t*-test and Pearson’s correlation coefficient. The interval between the two administrations was set at 7 days, in line with the recommendation that this interval should be long enough for respondents not to recall their previous answers, yet short enough for their status to remain unchanged; an interval of one to two weeks is generally considered adequate for this purpose. Differences in the influence of social media between two groups (sex) were tested using the independent-samples t-test, and between more than two groups (place of residence and nutritional status) using one-way analysis of variance (ANOVA). A two-sided *p*-value below 0.05 was considered statistically significant. Where the analysis of variance indicated a significant overall difference between groups, pairwise post-hoc comparisons were carried out in order to identify the groups that differed.

## 3 Results

### 3.1 Characteristics of the sample

The sample consisted of 362 participants, of whom 59.4% were female. The largest proportion of respondents lived in rural areas (47.8%) and the smallest in suburban areas (8.8%). The mean age of the respondents was 18.4 ± 0.59 years (Table 1). The mean self-reported body height was 174.1 ± 9.9 cm and the mean self-reported body weight was 70.3 ± 15.9 kg. According to BMI-for-age *z*-scores, 281 respondents (77.6%) were classified as having normal weight, 49 (13.5%) as overweight and 27 (7.5%) as obese, while 4 (1.1%) were classified as thin; data were missing for one respondent (0.3%). Overweight and obesity were more frequent among male respondents (33 and 17 respondents, respectively) than among female respondents (16 and 10 respondents, respectively).

**Table 1:** Characteristics of the study sample (*N* = 362).

Characteristic	Category	*n* (%) or mean ± SD
Sex	Female	215 (59.4)
	Male	147 (40.6)
Age (years)	Mean ± SD	18.4 ± 0.59
Place of residence	Urban	157 (43.4)
	Rural	173 (47.8)
	Suburban	32 (8.8)
School	Gymnasium	34 (9.4)
	Medical school	105 (29.0)
	Economics school	49 (13.5)
	Technical school	34 (9.4)
	Hospitality and trade school	24 (6.6)
	Traffic and electrical school	116 (32.0)
Body height (cm)^a^	Mean ± SD	174.1 ± 9.9
Body weight (kg)^a^	Mean ± SD	70.3 ± 15.9
Nutritional status^b^^,^^c^	Thinness (<−2 SD)	4 (1.1)
	Normal weight (−2 to +1 SD)	281 (77.6)
	Overweight (>+1 to +2 SD)	49 (13.5)
	Obesity (>+2 SD)	27 (7.5)
	Missing data	1 (0.3)

a Self-reported.

b BMI-for-age z-scores were calculated using WHO AnthroPlus software (WHO growth reference for children and adolescents aged 5–19 years) and classified according to age and sex.

c Distribution by sex: normal weight, 186 female and 95 male; overweight, 16 female and 33 male; obesity, 10 female and 17 male.

### 3.2 Transcultural adaptation and validation of the SESMEB questionnaire

#### 3.2.1 Factor analysis

In the statistical analysis of the data, Cronbach’s alpha coefficient, i.e., the average correlation between all values on the scale, was used to assess the internal consistency of the SESMEB questionnaire. In the first phase of statistical data processing, the research instrument was validated on a sample of 362 respondents. It was determined that the total Cronbach alpha coefficient was high enough (0.865) to conclude that the SESMEB questionnaire is a reliable research instrument. The suitability of the data for factor analysis was confirmed by the Kaiser-Meyer-Olkin measure of sampling adequacy (KMO = 0.857) and by Bartlett’s test of sphericity (*χ*^2^ (153) = 2312.380; *p* < 0.001). Using exploratory factor analysis, three subscales were determined that describe 51.5% of the variance. The first scale included seven questions, the second six and the third five questions of the questionnaire (Figure 1).

**Figure 1:**
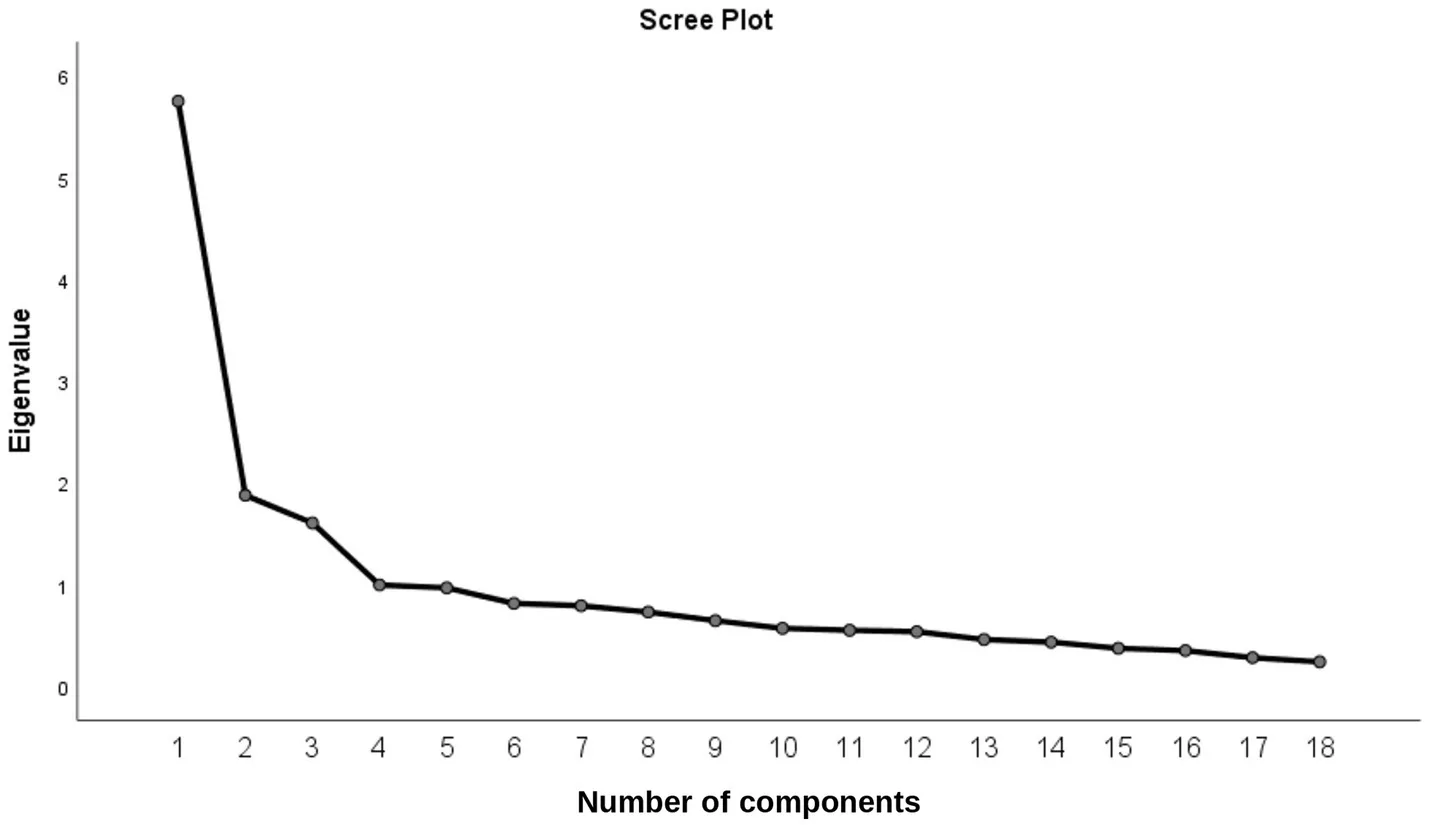
Scree plot of eigenvalues from the exploratory factor analysis of the 18-item SESMEB questionnaire.

#### 3.2.2 Testing the reliability and reproducibility of the questionnaire

The corrected Cronbach’s alpha coefficient for all items in the questionnaire was over 0.800, which was considered a satisfactory value during the reliability testing of the SESMEB questionnaire. Corrected item-total correlations ranged from 0.346 to 0.638. Two items, namely “I think that the food that appears on social media is healthier” (0.346) and “I follow news/blogs/pages about nutrition on social media” (0.385), fell below the threshold of 0.40 applied by the developers of the original instrument, while all remaining items exceeded this value (Table 2).

**Table 2:** Corrected item-total correlation of the SESMEB questionnaire.

Question	*α*	CITC*
1. Posting food on social media influences my opinion of that type of food.	0.860	0.438
2. I consume food that I see on social media even though it is not part of my usual eating habits.	0.858	0.481
3. Even though I am full, I eat the food/dish that I see on social media.	0.854	0.599
4. I think that the food that appears on social media is healthier.	0.863	0.346
5. Since I started using social media, I eat fast food more often.	0.857	0.500
6. I follow news/blogs/pages about nutrition on social media.	0.868	0.385
7. Without hesitation, I buy/cook the food/dishes that I see on social media.	0.861	0.420
8. I change my diet based on the news/pictures/videos about food that I see on social media.	0.860	0.441
9. I constantly eat snacks while browsing social media posts and only later realize how much I have eaten.	0.859	0.464
10. I am interested in the food that celebrities share on their social media profiles, and I consume that food/dish.	0.857	0.499
11. If I did not use social media, my time for eating would be less.	0.862	0.404
12. While I’m browsing social media, even though I’m full, I snack 5 times.	0.857	0.489
13. I eat food/dishes shared by people with a large number of followers on social media.	0.854	0.614
14. I think food/dishes with more likes/shares on social media are healthier.	0.856	0.494
15. Food/dishes I see on social media increase my desire to eat.	0.859	0.570
16. I eat food/dishes whose news/pictures/videos have more likes on social media.	0.852	0.563
17. I think food with more likes/shares on social media is more reliable.	0.851	0.466
18. On days when I use social media a lot, my desire to eat increases so I eat more.	0.851	0.638

*Corrected item-total correlation.

The reliability of the SESMEB questionnaire was carried out through the online form of the questionnaire, which the respondents filled out 7 days after the written version of the questionnaire (test–retest). Fourteen of initial respondents voluntarily agreed to participate in the retest survey and via SMS received an access link to fill out the questionnaire. After the identification of the respondents, statistical processing of the obtained data was performed (Table 3). The intraclass correlation coefficient (ICC) showed high reliability of our version of the SESMEB research instrument (>0.7). Using the t-test for paired samples, no statistically significant difference was found between the two tests (*t* = 0.552; *p* = 0.591). A satisfactory positive correlation was established between the scores of the first and repeated tests (Pearson’s coefficient = 0.699).

**Table 3:** Reproducibility testing of the SESMEB questionnaire.

Administration	Mean	SD	P25	P75	Test results
Test	30.43	9.04	22.51	39.25	*t* = 0.552, *p* = 0.591
Retest	31.43	6.92	23.75	36.50	ICC = 0.793; *r* = 0.699

*t*, *t*-test for paired samples; ICC, intraclass correlation coefficient; *r*, Pearson’s correlation coefficient; SD, standard deviation; P25, 25th percentile; P75, 75th percentile.

### 3.3 Assessment of the influence of social networks on the dietary habits of adolescents

#### 3.3.1 Social networks

All 362 respondents reported using at least one of the eight social networking platforms examined, and on average each respondent reported using 3.9 platforms. Instagram was the most widely used platform, reported by 354 respondents (97.8%), followed by Facebook (330; 91.2%), YouTube (257; 71.0%) and TikTok (241; 66.6%). Pinterest was used by just over one third of respondents (135; 37.3%), whereas Twitter (54; 14.9%), Snapchat (16, 4.4%) and LinkedIn (12, 3.3%) were used by comparatively few. The four most frequently used platforms were therefore those organized primarily around images and short video content (Table 4).

**Table 4:** Use of social networking platforms among respondents (*N* = 362).

Social networking platform	*n*	% of respondents	% of all reported uses
Instagram	354	97.8	25.3
Facebook	330	91.2	23.6
YouTube	257	71.0	18.4
TikTok	241	66.6	17.2
Pinterest	135	37.3	9.6
Twitter	54	14.9	3.9
Snapchat	16	4.4	1.1
LinkedIn	12	3.3	0.9
Total reported uses	1,399	—	100.0

All respondents reported using at least one of the eight platforms. Respondents could report more than one platform, so the percentages of respondents do not sum to 100; the final column expresses each platform as a proportion of all 1,399 reported platform uses. On average, respondents reported using 3.9 platforms. Platforms are listed in descending order of frequency of use.

#### 3.3.2 The influence of social networks on the habits, attitudes and practices of adolescent nutrition

More than 2/3 of adolescents (76.8%) stated that the number of likes/shares of a certain food does not affect their consumption of that food, and 69.9% of them do not consider such food more reliable. When we asked adolescents whether they consume food posted by people with a large number of followers, more than 3/5 of adolescents answered negatively and at the same time more than 2/3 of them (71.5%) do not consider such food healthier.

It was observed that in more than half of adolescents (55%), the time spent on social networks does not increase their desire to eat and does not affect uncritical food intake in almost 3/5 of adolescents (59.4%). What indicates the need for additional consideration of the results is the fact that 1/3 of adolescents (34.3%) stated that it is rare, and 1/5 (21.3%) that sometimes while viewing content on social networks, even though they are full, they still eat something, which is not negligible, and that 2/5 of respondents (42.4%) sometimes believe that food advertised on social networks is healthier (Table 5).

**Table 5:** The influence of social networks on the respondents’ attitudes and practices regarding the choice, preparation and consumption of food.

Question	*N* (%)				
	Never	Rarely	Sometimes	Often	Always
1. Posting food on social media influences my opinion of that type of food.	**139 (38.4)**	87 (24)	107 (29.6)	21 (5.8)	8 (2.2)
2. I consume food that I see on social media even though it is not part of my usual eating habits.	118 (32.6)	**130 (35.9)**	102 (28.2)	9 (2.5)	3 (0.8)
3. Even though I am full, I eat the food/dish that I see on social media.	**215 (59.4)**	88 (24.3)	46 (12.7)	9 (2.5)	4 (1.1)
4. I think that the food that appears on social media is healthier	86 (23.8)	118 (32.6)	**150 (41.4)**	8 (2.2)	0 (0)
5. Since I started using social media, I eat fast food more often	**144 (39.8)**	128 (35.4)	68 (18.8)	19 (5.2)	3 (0.8)
6. I follow news/blogs/pages about nutrition on social media.	100 (27.6)	**101 (27.9)**	**101 (27.9)**	49 (13.5)	11 (3.0)
7. Without hesitation, I buy/cook the food/dishes that I see on social media.	99 (27.3)	**134 (37.0)**	101 (27.9)	25 (6.9)	3 (0.8)
8. I change my diet based on the news/pictures/videos about food that I see on social media	**209 (57.7)**	102 (28.2)	42 (11.6)	8 (2.2)	1 (0.3)
9. I constantly eat snacks while browsing social media posts and only later realize how much I have eaten	**117 (32.3)**	110 (30.4)	83 (22.9)	39 (10.8)	13 (3.6)
10. I am interested in the food that celebrities share on their social media profiles, and I consume that food/dish.	**214 (59.1)**	76 (21)	53 (14.6)	17 (4.7)	2 (0.6)
11. If I did not use social media, my time for eating would be less.	**199 (55)**	81 (22.4)	57 (15.7)	17(4.7)	8 (2.2)
12. While I’m browsing social media, even though I’m full, I snack 5 times	**124 (34.3)**	**124 (34.3)**	77 (21.3)	26 (7.2)	11 (3)
13. I eat food/dishes shared by people with a large number of followers on social media.	**226 (62.4)**	89 (24.6)	39 (10.8)	7 (1.9)	1 (0.3)
14. I think food/dishes with more likes/shares on social media are healthier	**259 (71.5)**	67 (18.5)	27 (7.5)	7 (1.9)	1 (0.3)
15. Food/dishes I see on social media increase my desire to eat	95 (26.2)	91 (25.1)	**107 (29.6)**	47 (13)	22 (6.1)
16. I eat food/dishes whose news/pictures/videos have more likes on social media	**278 (76.8)**	48 (13.3)	28 (7.7)	7 (1.9)	1 (0.3)
17. I think food with more likes/shares on social media is more reliable	**253 (69.9)**	74 (20.4)	25 (6.9)	10 (2.8)	0 (0)
18. On days when I use social media a lot, my desire to eat increases so I eat more.	**199 (55)**	83 (22.9)	58 (16)	18 (5)	4 (1.1.)

Bold values indicate the most frequently reported response category for each item.

If we look at the results of the scale of the influence of social networks on eating behavior it can be concluded that social networks have no influence on the eating habits of the adolescents, because the score values for all the observed statements are found in the group with absolutely no influence or no influence (Table 6) according to the proposed classification of influence (Mean range = 1–1.8: absolutely no effect; Mean range = 1.81–2.60: does not affect).

**Table 6:** The influence of social networks on the eating habits of respondents.

Question	*N*	Min	Max	AS	SD
1. Posting food on social media influences my opinion of that type of food.	362	1	5	2.09	1.051
2. I consume food that I see on social media even though it is not part of my usual eating habits.	362	1	5	2.03	0.885
3. Even though I am full, I eat the food/dish that I see on social media.	362	1	5	1.62	0.880
4. I think that the food that appears on social media is healthier	362	1	4	**2.22**	0.833
5. Since I started using social media, I eat fast food more often	362	1	5	1.92	0.931
6. I follow news/blogs/pages about nutrition on social media.	362	1	5	**2.36**	1.114
7. Without hesitation, I buy/cook the food/dishes that I see on social media.	362	1	5	2.17	0.937
8. I change my diet based on the news/pictures/videos about food that I see on social media	362	1	5	1.59	0.801
9. I constantly eat snacks while browsing social media posts and only later realize how much I have eaten	362	1	5	2.23	1.121
10. I am interested in the food that celebrities share on their social media profiles, and I consume that food/dish.	362	1	5	1.67	0.930
11. If I did not use social media, my time for eating would be less.	362	1	5	1.77	1.021
12. While I’m browsing social media, even though I’m full, I snack 5 times	362	1	5	2.10	1.053
13. I eat food/dishes shared by people with a large number of followers on social media.	362	1	5	1.53	0.784
14. I think food/dishes with more likes/shares on social media are healthier	362	1	5	1.41	0.759
15. Food/dishes I see on social media increase my desire to eat	362	1	5	**2.48**	1.184
16. I eat food/dishes whose news/pictures/videos have more likes on social media	362	1	5	1.36	0.731
17. I think food with more likes/shares on social media is more reliable	362	1	4	1.43	0.742
18. On days when I use social media a lot, my desire to eat increases so I eat more.	362	1	5	1.74	0.972

Bold values indicate the three items with the highest mean scores, which were selected for the group comparisons presented in Tables 7–10.

The three items with the highest mean scores, namely “I think that the food that appears on social media is healthier”, “I follow news/blogs/pages about nutrition on social media” and “Food/dishes I see on social media increase my desire to eat”, were additionally examined in relation to sex, place of residence, nutritional status and the school attended. A statistically significant difference by sex was found for following nutrition-related news, blogs and pages (girls, *n* = 215, 2.56 ± 1.12; boys, *n* = 147, 2.07 ± 1.05; *p* < 0.05) and for the reported increase in the desire to eat (girls 2.58 ± 1.21; boys 2.33 ± 1.14; *p* = 0.048), whereas no difference was observed for the belief that food appearing on social media is healthier (girls 2.22 ± 0.83; boys 2.22 ± 0.84; *p* = 0.095) (Table 7).

**Table 7:** Mean scores on selected SESMEB items according to sex.

Item	Group	*n*	Mean	SD	*p*-value*
I think that the food that appears on social media is healthier	Female	215	2.22	0.830	0.095
	Male	147	2.22	0.840	
I follow news/blogs/pages about nutrition on social media	Female	215	2.56	1.117	**<0.05**
	Male	147	2.07	1.047	
Food/dishes I see on social media increase my desire to eat	Female	215	2.58	1.208	**0.048**
	Male	147	2.33	1.136	

*Independent-samples *t*-test. Statistically significant values (*p* < 0.05) are shown in bold.

With respect to place of residence, a statistically significant difference was found only for following nutrition-related content (city, *n* = 157, 2.45 ± 1.19; village, *n* = 173, 2.23 ± 1.01; suburban settlement, *n* = 32, 2.69 ± 1.20; *p* = 0.041) (Table 8). Post-hoc comparison showed that this difference lay between respondents living in villages and those living in suburban settlements, the latter using social networks for this purpose more frequently.

**Table 8:** Mean scores on selected SESMEB items according to place of residence.

Item	Group	*n*	Mean	SD	*p*-value*
I think that the food that appears on social media is healthier	Urban	157	2.31	0.807	0.184
	Rural	173	2.14	0.861	
	Suburban	32	2.19	0.780	
I follow news/blogs/pages about nutrition on social media	Urban	157	2.45	1.185	**0.041**
	Rural	173	2.23	1.012	
	Suburban	32	2.69	1.203	
Food/dishes I see on social media increase my desire to eat	Urban	157	2.48	1.180	0.990
	Rural	173	2.47	1.189	
	Suburban	32	2.50	1.218	

*One-way analysis of variance. Statistically significant values (*p* < 0.05) are shown in bold.

In relation to nutritional status, none of the three items differed significantly between groups (normal weight, *n* = 281; overweight, *n* = 49; obesity, *n* = 27; thinness, *n* = 4; all *p* > 0.05). Separate analysis of two further items showed a higher mean score among adolescents with obesity (2.70) than among those of normal weight (2.06) for the statement that posting food on social media influences their opinion of that type of food, and higher scores among adolescents with obesity (1.96) and thinness (2.50) than among those of normal weight (1.57) for eating food seen on social media despite being full. The latter comparison rests on only four respondents in the thinness group and should therefore be interpreted with caution (Table 9).

**Table 9:** Mean scores on selected SESMEB items according to nutritional status.

Item	Group	*n*	Mean	SD	*p*-value*
I think that the food that appears on social media is healthier	Normal weight	281	2.26	0.836	0.228
	Overweight	49	2.08	0.862	
	Obesity	27	2.07	0.730	
	Thinness	4	2.75	0.500	
I follow news/blogs/pages about nutrition on social media	Normal weight	281	2.39	1.129	0.779
	Overweight	49	2.22	1.104	
	Obesity	27	2.30	1.031	
	Thinness	4	2.50	1.000	
Food/dishes I see on social media increase my desire to eat	Normal weight	281	2.46	1.198	0.334
	Overweight	49	2.39	1.115	
	Obesity	27	2.74	1.228	
	Thinness	4	3.25	0.500	

*One-way analysis of variance. Nutritional status was classified from BMI-for-age *z*-scores as thinness (<−2 SD), normal weight (−2 to +1 SD), overweight (>+1 to +2 SD) and obesity (>+2 SD). Data were missing for one respondent. The thinness group comprised only four respondents, and comparisons involving this group should be interpreted with caution.

Comparison according to the school attended showed statistically significant differences for two of the three items (Table 10). Following nutrition-related news, blogs and pages differed between schools (*p* = 0.008), with the highest mean score recorded in the Gymnasium (*n* = 34, 2.71), followed by the Traffic and Electrical School (*n* = 116, 2.16) and the Technical School (*n* = 34, 1.97). The reported increase in the desire to eat also differed between schools (*p* = 0.004), with the highest score recorded in the Economics School (*n* = 49, 2.90), which differed significantly from the Gymnasium (2.35), the Traffic and Electrical School (2.32) and the Technical School (2.00). No difference between schools was found for the belief that food appearing on social media is healthier (*p* = 0.337).

**Table 10:** Mean scores on selected SESMEB items according to type of secondary school.

Item	Group	*n*	Mean	SD	*p*-value*
I think that the food that appears on social media is healthier	Gymnasium	34	2.09	0.793	0.337
	Medical School	105	2.19	0.773	
	Economics School	49	2.37	0.859	
	Technical School	34	2.00	0.953	
	Hospitality and Trade School	24	2.33	0.868	
	Traffic and Electrical School	116	2.27	0.838	
I follow news/blogs/pages about nutrition on social media	Gymnasium	34	2.71	1.292	**0.008**
	Medical School	105	2.59	1.071	
	Economics School	49	2.37	1.167	
	Technical School	34	1.97	1.218	
	Hospitality and Trade School	24	2.42	0.974	
	Traffic and Electrical School	116	2.16	1.012	
Food/dishes I see on social media increase my desire to eat	Gymnasium	34	2.35	1.228	**0.004**
	Medical School	105	2.56	1.064	
	Economics School	49	2.90	1.295	
	Technical School	34	2.00	1.128	
	Hospitality and Trade School	24	2.83	1.204	
	Traffic and Electrical School	116	2.32	1.177	

*One-way analysis of variance. Statistically significant values (*p* < 0.05) are shown in bold.

## 4 Discussion

With the advancement of technology, the media has become the most important source of information about health and nutrition. People increasingly make their daily decisions based on what they hear or see through the mass media (6). In their work, Aslan et al. state that the media has the greatest influence on body image and eating behavior in adolescents who are still unsure of their social reality, who are extremely sensitive to the opinions and messages of society, as well as information and stimuli from the environment that influence the shaping of their identity, since this is a period of intensive learning, development and identification (12). The constant infiltration of social media into our lives also requires research on their effects on the behavior of individuals related to nutrition. It is believed that research on the connection of social media as a dependent or independent variable and eating behavior is important for understanding the impact of content that is marketed through social media on eating attitudes and behaviors (8, 13). In 2015, Otrar and Argin (13) developed a scale that examined students’ attitudes toward social media in order to determine students’ attitudes toward this area of the Internet, but the shortcoming of this scale was that it did not examine eating behavior or the influence of social media on food attitudes. Guided by the fact that the national and international literature lacks a comprehensive, reliable and valid measuring instrument for examining the influence of social networks on eating behavior and attitudes, a group of authors developed and validated the SESMEB scale (Eng. Scale of effects of social media on eating behavior) in Ankara in 2020 (10), which initially contained 38 questions. In the process of developing the SESMEB questionnaire, the literature was first reviewed and the conceptual and theoretical basis of the characteristics to be measured by the mentioned research instrument was defined, and all items with a lower content validity ratio than 0.78 were excluded from the questionnaire, leaving 22. In the further development and validation of the SESMEB instrument, four more items with a corrected item-total correlation below 0.40 were excluded from the questionnaire, and the final version contained 18 questions. The version of the scale used in our research contained the same 18 items as the final version of the original questionnaire, whose single-factor structure was confirmed by confirmatory factor analysis in the original validation study (10). Exploratory factor analysis defined three subscales with 51.5% of the variance, but separate subscales could not be clearly separated with the assumption that the limiting factor is the similarity of questions that refer to identical statements. Cronbach’s alpha of our version of the questionnaire (0.865) was somewhat lower than the alpha of 0.928 reported for the original Turkish version (10), but both values indicate good internal consistency of the scale. Examining the reproducibility of our version of the questionnaire, it was determined that there is no statistically significant difference between the first and repeated measurements, which suggests that the questionnaire may be applied at more than one time point. This analysis, however, was based on only 14 respondents, and the absence of a statistically significant difference in such a small subsample cannot be taken as evidence of equivalence; the reproducibility of the Serbian version should therefore be regarded as preliminary and confirmed in a larger subsample. According to the results of researchers from Hungary, the Hungarian version of SESMEB is also valid and reliable and can be used to determine the influence of social media on eating behaviors. A limiting factor is the lack of a larger number of validation studies of the SESMEB questionnaire in order to enable its wider application and comparison of the obtained results between different countries (14).

In our research, we used the SESMEB questionnaire to assess the influence of social networks on the eating habits of adolescents and we showed that in more than half of the respondents (the range was from 55 to 76.8%), the influence of social networks on attitudes and practices related to the choice, preparation and consumption of food does not exist. About 76% of the respondents stated that the number of likes/shares of a certain food does not influence their consumption of that food, and 69.9% of them do not consider such food more reliable. More than 3/5 of respondents (62.4%) said that they do not eat food posted by people with a large number of followers, nor do they think that food is healthier (71.5%). It was observed that in more than half of the respondents (55%), the time spent on social networks does not affect the desire to eat, and in almost 3/5 of the respondents (59.4%) it was observed that social networks do not affect uncritical eating (taking food when full). Alwafi et al. came to a similar conclusion in a study conducted in Saudi Arabia in 2021, which included 1,124 respondents aged 18 and older. This study showed that social networks had an influence on the eating habits of only about one-fifth of the respondents (15). However, the results of research in Australia showed that there is a connection between the use of social networks and the number of profiles on social networks and eating disorders among adolescents (16). Similarly, a study in the United States of America (USA) conducted on a large cohort of eighth- and eleventh-grade students found that longer time spent on social media had a greater impact on poor diet and inadequate sleep in adolescents (17). In our research, 34.3% of the respondents stated that it is rare and 1/5 (21.3%) that sometimes while looking at content on social networks, even though they are full, they snack, which is not negligible, as well as that 2/5 of the respondents (42.4%) sometimes think that the food that appears on social networks is healthier. The results of a study conducted in Belgium, which included 1,002 students aged 11 to 19 in 18 secondary schools, showed that food messages and advertisements on social media are associated with adolescent diets. Students who reported greater exposure to posts of energy-dense and nutrient-poor foods, such as salty and sweet snacks, on social media were significantly more likely to consume higher amounts of these foods (18). The results of Coates et al.’s research on children’s exposure to food marketing on social networks are similar, where it was determined that children increase their intake of energy-dense and nutritionally poor foods when they are exposed to social media messages promoting these foods (19). An online questionnaire survey of 272 medical students in India showed a significant correlation between increased use of social media and the development of unhealthy eating habits, namely students who used 4 or more social networks were at greater risk of developing an eating disorder, as indicated by higher scores on the SESMEB. This was especially pronounced among first year students compared to final year students. First-year students were closer to the late adolescent age group, which is at the highest risk of developing an eating disorder. Going to medical school is first of all accompanied by leaving home and family, it implies leaving and living and eating in student canteens or switching to fast food due to a large number of obligations (20). In our research, female respondents reported following nutrition-related news, blogs and pages on social media more often than male respondents, and also reported a greater increase in the desire to eat in response to food content; both differences were statistically significant. In their survey of 1,000 students in Türkiye, Durmaz et al. showed that 81.1% of students are interested in the topic of nutrition on social media, that is, 31.2% of female students and 36.5% of male students are interested in and follow this type of content on social media (21). Also, the results of the study by Alwafi et al. indicate the probability that social networks affect women more (15), and this can be explained by gender differences in psychology and motives for using social networks, which Zhang et al. showed in their study (22). Research conducted in Indonesia among adolescent girls from 14 to 18 years old showed that exposure to social networks and time spent on social networks affects the choice of food and drink. The food and drink that the respondents chose was food and drink with a higher energy density, a higher proportion of fat and protein (23). Research by Tazeoglu et al., which examined the impact of social networks on appetite, food consumption, and body mass index, showed that young males view significantly more video content that includes food, as well as that males show increased appetite after watching a food video. Immediately after watching a video about food, male subjects were more likely to eat what they saw in the given content. When respondents were asked if their appetite increased after watching a food video, more than two-thirds of the participants answered yes (“yes”). Also, compared to women, men were significantly more likely to share food videos on their social media profiles and had more friends who further shared food videos with their followers. Although they showed that young girls use social networks significantly more, they actually thought significantly more about the calories they consumed and the amount of food they consumed. Also, the authors saw a clear correlation between BMI and the length of time spent on social networks, spent viewing food videos, primarily due to reduced movement and excessive food intake while sitting in front of a screen. Therefore, we can say that social networks can really influence body mass, given that social norms encourage the consumption of certain foods, and that this influence differs depending on the gender of young people (24). In a study by Temple et al., more than half of respondents (63%) did not consume food advertised on television. This could be related to fewer hours of television viewing and therefore less exposure to food advertisements on television. When adolescents were exposed to more hours of television viewing, there was also an increase in the consumption of advertised foods (25). A study by Cheah et al. showed that a higher percentage of respondents used cereals as opposed to unhealthy foods, such as soft drinks and energy-dense, nutritionally poor foods (26). This result differed from the results of a study conducted in the United States of America, which showed that the consumption of soft drinks while watching television was higher (27).

In contrast to us, Sina et al. concluded in their systematic literature review that exposure to social media was associated with increased frequency of consumption of snacks, fast food, sweetened juices and higher odds of skipping breakfast, with those living in rural and suburban areas being at greater risk (28). In relation to the secondary school attended, our results showed statistically significant differences for two of the three items examined: adolescents attending the Gymnasium reported following nutrition-related content most frequently, whereas those attending the Economics School reported the greatest increase in the desire to eat in response to food content.

Kim and Han showed that adolescents in Korea who use smartphones for communication rather than for learning are more likely to consume fast food (29). Teo et al. showed that adolescents who use WhatsApp for messaging consume 58% more snacks than adolescents who read an article published online (30). Gascoyne et al. found that 55% of students reported seeing an advertisement for food or drink on social media at least once a week, although consuming the advertised products was less frequent; exposure to such advertisements at least once in the previous week was associated with a high intake of unhealthy beverages, while liking or sharing food or beverage posts at least once in the previous month was associated with a high intake of both unhealthy beverages and unhealthy foods, and this association was stronger in boys (31). A study conducted in Indonesia in 2021 among 123 respondents examined the relationship between eating behavior, frequency and duration of social media use, and total consumption of energy-dense foods, and found that both emotional overeating and time spent on social networks were associated with a higher intake of energy-dense foods (32). Social media use can influence food choices through food advertising and through the influence of other users, as social networks allow advertising to spread quickly and reach large numbers of consumers (33). Kucharczuk and colleagues reported two main findings: adolescents recalled unhealthy foods more often and were influenced by celebrities and influencers, suggesting that food and beverage companies use social media to target the adolescent population (34). Niškanović et al. (35) piloted the “L: Landscape of campaigns” step of the WHO “CLICK” framework in the Republic of Srpska, Bosnia and Herzegovina, to characterize the digital food and beverage marketing to which children are potentially exposed through their preferred digital media. The most frequently advertised food categories on social media and in YouTube influencers’ content were cakes, sweet biscuits and pastries (31 and 22.7%, respectively) and chocolate and sugar confectionery (27.5 and 14.7%, respectively). None of the social media advertisements, and only 2 of 24 YouTube influencer marketing instances, would have been permitted to be marketed to children, and most advertisements featured elements potentially appealing to teenagers (88% on Facebook and 72% on Instagram). The most frequent persuasive appeals on social media were references to holidays, travel or adventure (23%), taste (12.5%) and premium/contest offers (11.5%), while YouTube influencers predominantly presented branded foods (62.5%). In line with these observations, our study showed that adolescent girls followed more food-related content and that such content increased their desire to eat.

Taken together, these findings should be interpreted in the light of what the SESMEB scale actually measures. The instrument assesses the influence of social media on eating behavior as perceived and reported by respondents themselves, and perceived influence is not equivalent to actual influence. People systematically judge advertising to affect others more than themselves, and recognition of persuasive intent is weakest precisely for influencer and native content, which does not present itself as advertising; influence that operates through repeated exposure, affect and perceived social norms is therefore largely unavailable to introspection. The pronounced floor effect observed for several items, with between 60 and 77% of respondents selecting the response “never,” is consistent with this interpretation. The contrast with the marketing landscape documented for the same population is instructive: while the adolescents in our sample reported almost no influence, an audit of the digital media they prefer showed intensive marketing of energy-dense, nutritionally poor products, with 88% of advertisements on Facebook and 72% on Instagram containing elements potentially appealing to teenagers (35). Our results should therefore not be read as evidence that social media does not influence the eating behavior of adolescents, but rather that adolescents do not recognize such an influence, which is in itself relevant for the design of media literacy interventions and for the choice of measurement approaches in future research.

## 5 Limitations and strengths

This study has several limitations and we can consider it a pilot study for the area of Bosnia and Herzegovina, but at the same time it has its advantages since it is a very current topic and this is the first step in considering this aspect of the influence on the eating habits of young people. In terms of strengths, this study presents the first efforts to evaluate the validity and reliability of the SESMEB questionnaires which is easy to use, cost-effective, and reproducible.

The first limitation is reflected in the fact that it was conducted only in one geographical area and that the results cannot be applied to the whole of Bosnia and Herzegovina.

Second, the questionnaire has so far been applied and validated in a smaller number of countries, and the results cannot be compared and monitored.

Third, the lack of validated versions of this questionnaire that have been used in other countries and in other languages, which would allow for comparison of the results obtained with other countries, is also considered a limiting factor.

Fourth, the absence of questions that examine the frequency and duration of social media use, as well as the focus on the content that respondents use, which may influence their answers, can be also recognized as a limitation of the study and the instrument used in the study.

Fifth, body height and body weight were self-reported rather than measured, which may have led to misclassification of nutritional status. Sixth, the test–retest subsample comprised only 14 respondents, so the reproducibility estimates should be regarded as preliminary. Seventh, measurement invariance across sex, place of residence and nutritional status was not tested, and the comparisons between these groups should therefore be interpreted with corresponding caution. Finally, several items showed a pronounced floor effect, with between 60 and 77% of respondents selecting the response “never,” which limits the variability available for detecting differences between groups. In addition, the design does not allow the two pathways through which social media may affect eating behavior, namely the effect on consumption decisions and the shaping of social norms, to be separated empirically, since the instrument measures perceived influence and contains no measure of actual exposure, of the frequency or duration of use, or of the type of content followed.

## 6 Conclusion

As colleagues from Türkiye concluded, there is no comprehensive tool in the literature for measuring the impact of social media on eating habits. The SESMEB Questionnaire proposed by them, which was tested in our research, can be applied as an adequate instrument for measuring the impact of social media use on eating habits. Our research also indicated the need to correct certain questions in order to get more reliable answers to certain questions. Certainly, the use of the mentioned instrument by a larger number of authors in several countries would give an opportunity for a better understanding of its application and its improvement, since it represents an important contribution in the field of considering the effect of social media on eating habits. The results of our study represent the basis for conducting future studies on the mentioned topic in our area in order to gain insight into the influence of social networks on the eating habits of adolescents in different geographical areas in Bosnia and Herzegovina and to be able to undertake interventions. Future research may explore whether the type of food largely advertised varies depending on adolescents’ nutrition knowledge and interests (for example, whether adolescents who choose to follow an exercise regimen or implement a particular diet receive more or less fast-food and HFSS foods advertisements).

Two practical implications follow from these results. First, adolescents reported almost no influence of social media on their eating behavior, although the same population is intensively exposed to digital marketing of energy-dense, nutritionally poor products. Preventive work should therefore be directed toward media literacy, and specifically toward the recognition of persuasive intent in influencer and native content, rather than toward nutritional knowledge alone. Second, the differences observed between schools indicate that school-based programs should not be uniform, and that the school context is a relevant unit for the planning of preventive activities.

## Data Availability

The original contributions presented in the study are included in the article/supplementary material, further inquiries can be directed to the corresponding author.
